# Trends in Drug Overdose Mortality in Ohio During the First 7 Months of the COVID-19 Pandemic

**DOI:** 10.1001/jamanetworkopen.2021.7112

**Published:** 2021-04-14

**Authors:** Janet M. Currie, Molly K. Schnell, Hannes Schwandt, Jonathan Zhang

**Affiliations:** 1 Center for Health and Wellbeing, Princeton University, Princeton, New Jersey; 2 National Bureau of Economic Research, Cambridge, Massachusetts; 3 Department of Economics, Northwestern University, Evanston, Illinois; 4 School of Education and Social Policy, Northwestern University, Evanston, Illinois

## Abstract

This cross-sectional study uses data from the Ohio Department of Health to evaluate trends in drug overdose mortality in that state by type of drug and user age during the first 7 months of the COVID-19 epidemic.

## Introduction

The COVID-19 pandemic has been associated with excess deaths relative to existing trends.^1^ Because drug overdoses are a leading cause of death, it is important to investigate the general time-series pattern of overdose deaths during the pandemic. While there have been reports of increased overdoses in 2020,^2^ other evidence suggests that overdose deaths, especially those due to fentanyl, were increasing before the pandemic.^3^ Because national data lag data available at the local level, the use of local data is important to examine how closely increases in overdose deaths in 2020 tracked the course of the pandemic.

## Methods

All 12 195 overdose deaths in Ohio (8140 males [66.7%]) from January 1, 2018, through October 10, 2020, were examined using publicly available data from the Ohio Department of Health.^4^ Fatal overdoses were classified by drug type as in previous research^5^ and were plotted for each week. Overdose deaths for 4 age groups (18-24 years, 25-44 years, 45-64 years, and 65 years and older) in each 4-week period were compared with the average number of deaths in each group in 2018 through 2019. Under the Common Rule (82 FR §7149), there is no need for patient consent or institutional review board approval because the data were publicly available and included no identifiable patient information. All data analyses were performed using R version 4.0.2 (R Core Team). This cross-sectional study followed Strengthening the Reporting of Observational Studies in Epidemiology (STROBE) reporting guideline.

## Results

**Figure 1.**
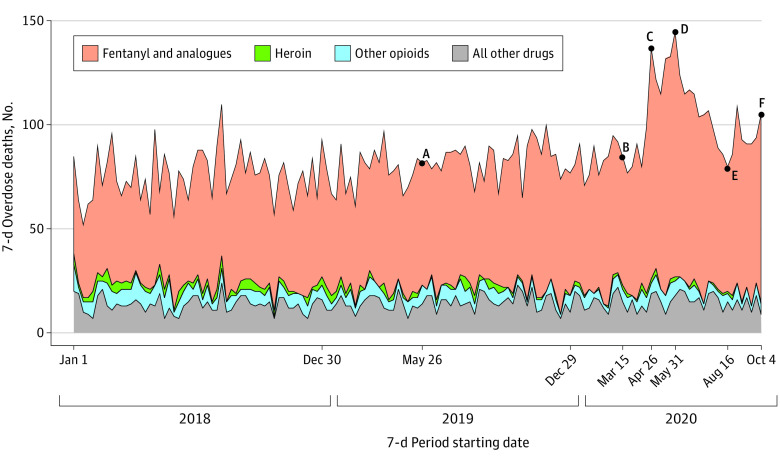
Weekly Overdose Deaths, January 1, 2018, to October 10, 2020, by Drug Type Overdose deaths from medical examiners’ offices are shown for all Ohio counties. At point A (May 26 to June 1, 2019), there were 82 overdoses (59 fentanyl and analogues, 0 heroin, 9 other opioids, and 14 other drugs). At point B (March 15-21, 2020), there were 85 overdoses (62 fentanyl and analogues, 2 heroin, 6 other opioids, and 15 other drugs). At point C (April 26 to May 2, 2020), there were 137 overdoses (111 fentanyl and analogues, 2 heroin, 5 other opioids, and 19 other drugs). At point D (May 31 to June 6, 2020), there were 145 overdoses (118 fentanyl and analogues, 2 heroin, 7 other opioids, and 18 other drugs). At point E (August 16-22, 2020) there were 80 overdoses (60 fentanyl and analogues, 1 heroin, 5 other opioids, and 9 other drugs). At point F (October 4-11, 2020), there were 105 overdoses (90 fentanyl and analogues, 1 heroin, 5 other opioids, and 9 other drugs). The “other opioids” category included nonfentanyl, nonheroin opioids.

In all, 12 195 overdose deaths occurred in Ohio from January 1, 2018, through October 10, 2020. Of these, 8140 (66.7%) were men. Figure 1 shows weekly drug overdose deaths by drug type during the period studied. Fatal overdoses rose sharply, from 85 in the week following the declaration of a national emergency (point B) to a peak of 145 overdoses in the week of May 31 (point D)—an increase of 70.6%. This peak represents an increase of 76.8% relative to the 82 fatal overdoses in the same week a year earlier (point A). Fatal overdoses fell to 80 by mid-August (point E) and rose to 105 in the last sample week. The 8981 fentanyl-related deaths represented 73.6% of total fatal overdoses and were the only drug category that spiked over the sample period. The “other opioids” category included nonfentanyl, nonheroin opioids.

**Figure 2.**
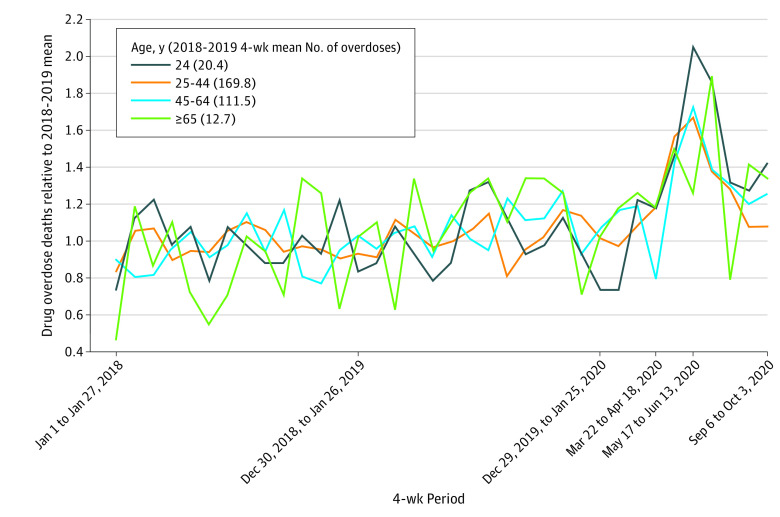
Overdose Deaths in Each 4-Week Period by Age, Normalized by 2018-2019 Average, January 1, 2018, to October 10, 2020 Overdose deaths from medical examiners’ offices are shown for all Ohio counties. Overdose death counts are aggregated into 4-week periods for 4 age groups. Levels in each 4-week period are divided by the overall mean number of overdoses in the relevant age group for 2018 through 2019.

Figure 2 shows overdose deaths in each 4-week period from January 1, 2018, through October 10, 2020, relative to the mean for 2018-2019 in each age group. Overdose deaths increased and then decreased in all 4 age groups, with the largest relative spike occurring among the youngest group. For those aged 24 years and younger, 4-week overdoses at the peak were 2.06 times the 2018-2019 four-week mean (42 vs 20.42 deaths). For those aged 25 to 44 years, 4-week overdose deaths peaked at 1.67 times the 2018-2019 four-week mean (284 vs 169.77 deaths); for those aged 45 to 64 years, 1.72 times (192 vs 111.46); and for those aged 65 or older, 1.89 times (24 vs 12.69).

## Discussion

In this cross-sectional study, the temporal pattern of overdoses followed some features of the COVID-19 pandemic, albeit with some time lag. An initial spike in overdose deaths followed the declaration of a national public health emergency on March 13, 2020; early state and local lockdowns and restrictions; and an Ohio unemployment rate that hit 17.6% by April 2020. By August 2020, lockdowns and restrictions had eased, and Ohio’s unemployment rate had fallen to 8.9%. Overdose deaths also returned to levels within recent historical experience.

The initial spike in deaths was most pronounced for the youngest adults, consistent with large self-reported deteriorations in their mental health and increased drug use.^6^ However, fatal overdoses followed a similar pattern in all age groups considered, including those 65 years or older.

One limitation of our study is that the cause of death is still pending for 0.034% of deaths (121 of 351 834 total deaths) over our sample period. We are therefore missing a small number of deaths that could eventually be counted as overdoses. In addition, our findings may not be generalizable outside the state of Ohio. We found similar results in several large counties that provided medical examiner data, but it is not yet possible to conduct a similar analysis using a nationally representative sample. Still unknown is whether and how the pandemic caused this spike in fatal overdoses, and why overdose deaths returned to baseline levels after rising sharply at the start of the pandemic.
