# Errors in Methods Section and Figure Key

**DOI:** 10.1001/jamanetworkopen.2021.14947

**Published:** 2021-05-21

In the Research Letter titled “Trends in Drug Overdose Mortality in Ohio During the First 7 Months of the COVID-19 Pandemic,”^1^ published April 14, 2021, there were errors in the presentation of the youngest age category in the text and a figure. In the Methods section, the age range of the youngest category should have appeared as “0-24.” In the key to Figure 2, the top age category should have appeared as “≤24.” This article has been corrected.^1^
